# Rosiglitazone and bezafibrate modulate gene expression in a rat model of non-alcoholic fatty liver disease - A historical prospective

**DOI:** 10.1186/1476-511X-12-41

**Published:** 2013-03-25

**Authors:** Hemda Schmilovitz-Weiss, Edith Hochhauser, Michal Cohen, Yelena Chepurko, Smadar Yitzhaki, Ehud Grossman, Avshalom Leibowitz, Zvi Ackerman, Ziv Ben-Ari

**Affiliations:** 1 Division of Gastroenterology, Rabin Medical Center, Hasharon Hospital, Petach Tikva, Israel and Sackler School of Medicine, Tel Aviv University, Tel Aviv, Israel; 2 Liver Institute, Rabin Medical Center, Beilinson Hospital, Petach Tikva, Israel and Sackler School of Medicine, Tel Aviv University, Tel Aviv, Israel; 3 Cardiac and Liver Research Laboratory, Felsenstein Medical Research Center, Petah Tikva, Israel; 4 Faculty of Life Sciences, Bar-Ilan University, Ramat Gan, Israel; 5 Department of Internal Medicine D and Hypertension Unit, Chaim Sheba Medical Center, Tel Hashomer, and Sackler School of Medicine, Tel Aviv University, Tel Aviv, Israel; 6 Department of Medicine, Hadassah University Hospital on Mount Scopus, Jerusalem, Israel

**Keywords:** Bezafibrate, Rosiglitazone, Hepatic steatosis, Fructose enriched diet

## Abstract

**Background:**

Genetic factors implicated in the pathogenesis of non-alcoholic fatty liver disease are poorly understood. Our aim was to characterize three genes involved in a rat model of non-alcoholic fatty liver disease and investigate the effect of rosiglitazone and bezafibrate.

**Method:**

Five rats were fed a chow diet (controls) and 18 a fructose-enriched diet (FED) for 5 weeks: 6 were administered rosiglitazone and 6 bezafibrate during the last 2 weeks and 6 were not treated at all. Livers were examined by reverse transcription-PCR for the genes encoding peroxisome proliferator-activated receptors (PPAR), PPAR-α, PPAR-γ, and Mn superoxide dismutase2 (Mn SOD2). Western blot was used for proteins levels.

**Result:**

The FED rats showed a decrease in mRNA of MnSOD2, PPAR-α, and PPAR-γ (3, 3.5 fold, and 27%, respectively) (p<0.05). The 3 genes normalized in response to rosiglitazone and bezafibrate. The proteins of MnSOD2, PPAR-α and PPAR-γ in the FED rats decreased (2.5, 2, and 2.2, respectively) (p<0.05). Following administration of rosiglitazone, proteins of MnSOD2, PPAR-α and PPAR-γ in the FED rats increased (reaching 1.5-fold, a 20% increase and normalization, respectively), (p<0.05). Administration of bezafibrate to the FED rats restored the proteins of 3 genes to baseline.

**Conclusion:**

A consistent reduction in hepatic expression of MnSOD2, PPAR-α and PPAR-γ in the FED rats compared with controls was observed. Administration of either rosiglitazone or bezafibrate to the FED rats restored these genes to a pre-morbid state.

## Introduction

Nonalcoholic fatty liver disease (NAFLD) is considered the hepatic manifestation of the metabolic syndrome [1]. The clinical spectrum of NAFLD ranges from simple steatosis to steatohepatitis, bridging fibrosis, and cirrhosis [2]. The primary event of NAFLD is the accumulation of triglycerides in hepatocytes which appear to be determined by insulin resistance [3]. The secondary event is hepatocellular injury (nonalcoholic steatohepatitis - NASH) involving factors such as oxidative stress, increased proinflammatory cytokines, mitochondrial dysfunction, iron overload, bacterial overgrowth and genetic predisposition [4]. Although metabolic risk factors for NAFLD progression are well known, the role of genetic susceptibility remains unclear [5-7].

Recent studies have implicated the nuclear hormones peroxisome proliferator-activated receptors (PPARs), PPAR-α and PPAR-γ in the pathogenesis of NAFLD [8-12]. PPAR-α reduces liver fat by increasing β-oxidation of fatty acids. PPAR-γ increases insulin sensitivity, thus reducing fatty acid flux to the liver. Accordingly, PPAR ligands have shown promise in the treatment of NAFLD [13-26].

Rosiglitazone has a high binding affinity with PPAR-γ [8,11,13-16,18,19]. Fibrates such as bezafibrate exert their effects by activating PPAR-α [8-26]. The oxidative stress induced by steatosis is counteracted by cellular enzymatic and nonenzymatic antioxidant systems. The cytosolic and mitochondrial superoxide dismutases (SODs), in addition to glutathione peroxidase and catalase, eliminate the reactive oxygen species (ROS) formed during the fatty acid oxidation process [27].

Non-obese rats fed a fructose-enriched diet (FED) have been found to acquire histologically-proven NAFLD and also exhibit certain aspects of the metabolic syndrome, such as hypertension, insulin resistance, and hyper-triglyceridemia [28].

In this study, samples archived from previous studies from 2005 [28] and 2007 were analyzed [29]. Changes in plasma and hepatic triglycerides and phospholipids have been previously published in response to administration of rosiglitazone and bezafibrate [28,29]. Histological changes were assessed by modifying the scoring system of the grading and staging of non-alcoholic steatohepatitis as described by Brunt et al. [30]. The histological evaluation of the liver sections was blindly performed [29].

The livers of the control group (rats fed with a standard rat chow diet, Koffolk) showed no signs of macrovesicular steatosis or fibrosis. The livers of the FED rats showed mild to moderate macrovesicular and microvesicular fat with minimal signs of perisinusoidal fibrosis.

The FED rats had 188% higher plasma triglyceride levels compared to the control group. Changes in liver enzymes were not observed between groups, however, the ratio of alanine to aspartate aminotransferase which was 1.2:1.0 in the control group increased to 2.0:1.0 in the FED rats. The livers of the FED rats had higher concentrations of total lipids (+28%), triglycerides (+198%), and cholesterol (+89%), but lower concentrations of phospholipids (−36%) compared to the livers of the control rats.

Administration of rosiglitazone to the FED rats was associated with decreases in plasma triglycerides (−62%), hepatic total lipids (−19%), hepatic triglycerides

(−61%), and increases in hepatic phospholipids (+46%). Administration of rosiglitazone to the FED rats initiated a decrease in the hepatic macrovesicular steatosis score without an improvement in the relative fibrosis area [29]

Administration of bezafibrate caused a reduction in plasma (−49%) and hepatic (−78%) triglycerides concentrations and an increase in hepatic phospholipids (+41%) [28]. Administration of bezafibrate resulted in a significant reduction in the hepatic macrovesicular steatosis but no change in the microvesicular steatosis, inflammatory, or fibrosis score [28].

The aim of this study was to characterize the expression of principle genes encoding enzymes involved in fatty acid metabolism (MnSOD2, PPAR-α and PPAR-γ) in a rat model of NAFLD and record the changes in their levels following administration of either rosiglitazone or bezafibrate.

## Material and methods

### Animals and experimental protocol

This study was performed on 23 male Sprague–Dawley rats (Harlan Laboratories Ltd., Jerusalem, Israel) weighing 200–280 grams, housed in regular cages, situated in an animal room at 22°C and maintained on a 14-hour light/10-hour dark cycle. The rats were randomly divided into 4 groups. One group, serving as controls, (n=5) was maintained on a standard rat chow diet for 5 weeks (Koffolk, Tel Aviv, Israel) with tap water ad libitum. The other 3 groups (6 rats in each) were given FED for 5 weeks. Three weeks after commencement of FED, one FED group was also given rosiglitazone (10 mg /kg/day) and another group, bezafibrate (10 mg/kg/day), for an additional 2 weeks. Drugs were administered via the drinking water.

All rats were sacrificed after 5 weeks and their livers excised for laboratory analysis. All animal studies were conducted according to the regulations for the use and care of experimental animals (National Institutes of Health Guidelines, USA). Our study used archived specimens from previously conducted studies from 2005 [28] and 2007 [29]. Upon termination of each experiment (animals sacrifice), liver samples of the rats were cut into small sized sections to be preserved either as frozen material (−70°C) or in formalin and as paraffin embedded slices. None of the frozen liver specimens had been subjected to thawing and refreezing. These specimens were still viable for analysis through molecular biology techniques.

All rats were sacrificed after 5 weeks and their livers excised for laboratory analysis. All experiments were carried out in accordance with the guidelines of the Animal Care and Use Committee of the Chaim Sheba Medical Center, Israel

### Western blot analysis of protein expression

Liver tissue samples (20 mg per sample) from the 23 rats were homogenized in a lysis buffer and quantified for protein levels using a commercial assay (Bio-Rad, USA). Proteins (50 μg/sample) were separated using SDS-polyacrylamide gels (12.5%) under denaturing conditions and electrotransferred onto nitrocellulose Bio-Rad) for 1 hour at 100 V.

Membranes were blocked with 5% nonfat milk in a tris-buffered saline (TBS) containing 0.1% tween 20 (TBST). The primary antibodies, anti-PPAR-γ, anti-PPAR-α, anti-SOD (Santa Cruz Biotechnology, Santa Cruz, CA), and anti-β-actin (Sigma, St. Louis, MO) were diluted 1:1000 in TBST with 5% nonfat milk and left overnight at 4°C. After 3 washes in TBST, the membranes were incubated with the secondary antibody for 30 to 60 minutes, as appropriate. Immunodetection and optical density measurement of the appropriately sized bands were performed using the Odyssey infrared imaging system (LI-COR Biosciences, Cambridge, UK).

### Quantitative reverse-transcriptase polymerase chain reaction (RT-PCR)

RNA was isolated from the rat livers by mechanical homogenization and extraction in TRIzol. Reverse transcription was performed using the Applied Biosystems High Capacity cDNA Reverse Transcription Kit (Kit 4374966; Ambion/Applied Biosystems, Austin, TX, USA). cDNA concentrations were determined with a Nanodrop spectro-photometer (Nanodrop Technologies, Wilmington, DE 19810 United States) followed by absolute quantitative real-time PCR performed with the 9700HT instrument (Applied Biosystems, Foster City, CA) set to the default 40-cycle program. TaqMan gene expression assays were purchased from Applied Biosystems: PPAR-α, Mm0481934; PPAR-γ, Mm00713303; SOD1 4352932E. Expression levels were normalized to the TATA box binding protein (TBP) reference gene (assay Mm00446973_m1). For each amplicon, PCR efficiency was estimated to be near 1.0 by serial dilutions of cDNA. Relative quantities of mRNA were estimated using the delta CT method.

### Setting

The rats were handled at the Hypertension Research Laboratory of the Chaim Sheba Medical Center, Tel-Hashomer and their livers were kindly donated by E. Grossman (co-author) [31]. Z. Ackerman (co-author) characterized the rats clinically and histopathologically [28,29]. All experiments were carried out in accordance with the guidelines of the Animal Care and Use Committee of the Chaim Sheba Medical Center, Israel**.**

The analyzed samples in the current study were archived from previous studies from 2005 [28] and 2007 [29]. The stored frozen and formalin fixed liver tissues were processed for gene expression analysis at the cardiac and liver Research Laboratory, Felsenstein Medical Research Center, Petah Tikva, Israel.

### Statistical analysis

Data are given as means ± standard deviations. Statistically significant differences in mean values of the variables between the groups were assessed by one way analysis of variance (ANOVA). If differences were found, values were then compared using the Student’s t-test, p < 0.05 was considered significant.

## Results

### MnSOD2

**Figure 1:**
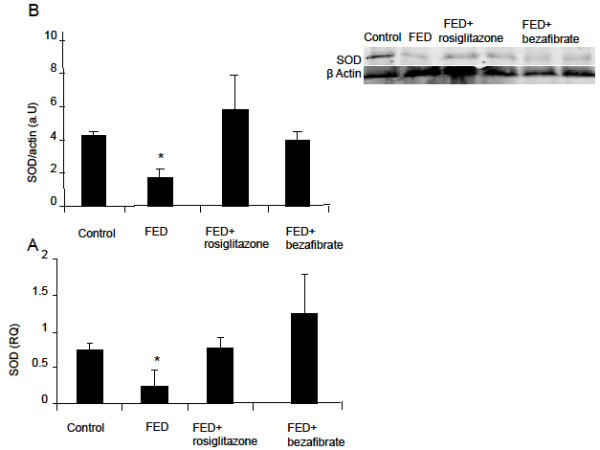
**Effect of rosiglitazone and bezafibrate on MnSOD2 gene expression (A) and protein level (B).** * = p<0.05.

Hepatic MnSOD2 mRNA expression decreased 3-fold in the FED rats compared with the rats given standard rat chow (controls) (p<0.05). The hepatic protein level of MnSOD2 decreased 2.5-fold compared to the controls (p<0.05). Following administration of rosiglitazone, gene expression returned to baseline and protein level reached a 1.5-fold of baseline (control) level (p<0.05). MnSOD2 mRNA expression and protein level were restored to baseline level in the bezafibrate-treated rats (Figure 1A and 1B).

### PPAR-α

**Figure 2:**
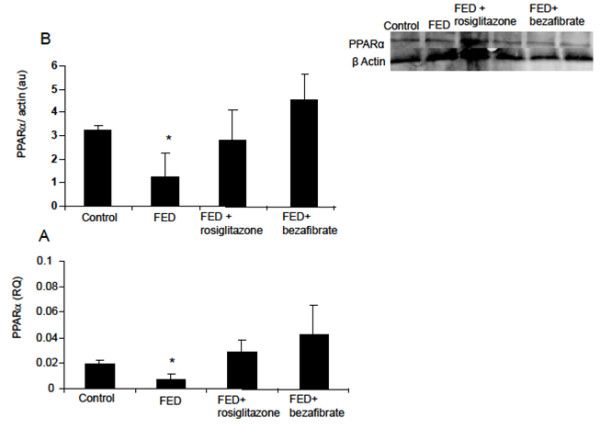
**Effect of rosiglitazone and bezafibrate on PPAR- α gene expression (A) and protein level (B).** * = p<0.05.

Hepatic PPAR-α mRNA expression decreased 3.5-fold in the FED rats compared with the control levels (p<0.05). The hepatic PPAR-α protein level, decreased 2-fold (p<0.05). Administration of rosiglitazone restored both gene expression and protein level to baseline; bezafibrate treatment restored both gene expression and protein level to baseline (control level) (Figure 2A and 2B).

### PPAR-γ

**Figure 3:**
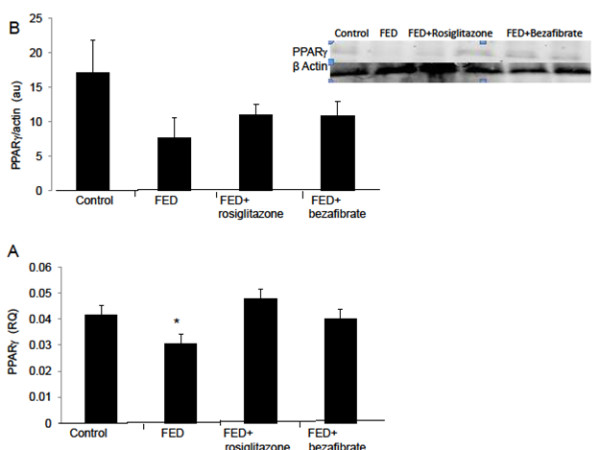
**Effect of rosiglitazone and bezafibrate on PPAR- γ gene expression (A) and protein level (B).** * = p<0.05.

Hepatic PPAR-γ mRNA expression decreased by 27% in the FED rats compared with the controls levels (p<0.05). Hepatic protein level decreased 2.2-fold. Rosiglitazone and bezafibrate administration restored gene expression to baseline. Protein levels reached 20% above the control levels (p<0.05) in the rosiglitazone treated group and reached control levels in the bezafibrate-treated rats (Figure 3A and 3B).

## Discussion

The present study demonstrates a statistically significant downregulation of hepatic MnSOD2, PPAR-α and PPAR-γ mRNA expression in FED rats with NAFLD compared with controls. Following administration of either rosiglitazone or bezafibrate, the expression of all three genes normalized. A statistically significant decrease in hepatic protein levels of all three genes in the FED rats compared with controls was also demonstrated. Administration of both rosiglitazone and bezafibrate to the FED rats restored to baseline or increased the levels of all three proteins.

Kohjima et al. [32] reported a reduction in PPAR-α expression but no change in PPAR-γ expression in cirrhotic patients with NAFLD.

Earlier studies reported a marked increase in PPAR-γ expression in fatty livers [20,21] as to the mRNA level and function of the related protein. Conversely, adipogenesis resulting in triglyceride storage occurred under conditions of decreased PPAR-α activity and fatty acid oxidation [22], implying cross-talk between PPAR-γ and PPAR-α.

Our results are consistent with reports of a loss of PPAR-α gene expression in mice with hepatic steatosis under fasting or high-fat diet conditions [10,17] and a decrease in hepatic steatosis in mice on a methionine- and choline- deficient diet after administration of a potent PPAR agonist [8,18,19]. These observations indicate that in the presence of increased hepatic fatty acid influx or decreased hepatic fatty acid efflux activation, PPAR-α prevents accumulation of triglycerides by increasing the rate of fatty acid catabolism.

Our results are in accordance with Tipoe et al. [33] who studied rats fed on a high-fat diet, and found significantly decreased SOD1 and SOD2. This effect was reversed by pioglitazone co-administration. In contrast, Kohjima et al. [32] observed a 5-fold increase in SOD expression in human NAFLD. Sreekumar et al. [34] however, as in the present study, found a decreased expression of SOD1 in cirrhotic livers of patients with NASH.

Videla et al. [35] correlated hepatic and plasma oxidative stress-related parameters with clinical and histological findings in 31 patients with NAFLD, finding that liver SOD activity decreased in the entire group by 48% compared with the controls (p<0.05) and by 64% in patients with steatohepatitis (n=16) (p<0.05). The authors concluded that oxidative stress develops in the livers of patients with steatosis and is exacerbated in patients with steatohepatitis associated with Cytochrome P450 2E1 (CYP2E1) induction. The substantial protein oxidation followed by proteolysis of the modified proteins could account for the coexistence of diminished antioxidant capacity and protein oxidation in the liver of patients with steatohepatitis.

Most of the data on PPAR-α and hepatic lipid homeostasis originates from rodent models. There are, however, important differences in PPAR-α activity between rodents and humans. PPAR-α DNA binding activity and PPAR-α expression in human hepatocytes is 10-fold less than observed in mice [36-38]. In addition, certain PPAR response elements do not respond to PPAR ligands in humans as they do in rodent models.

It may be argued that apart from species differences between human and rodent hepatic genes, the contradictory results among studies may be explained by differences in the degree of damage to the fatty liver. Our rats were sacrificed on the 35th day of the study, and the only overt histological finding was macrovesicular steatosis which was mitigated in the treated animals [28,29].

In the present study, administration of either rosiglitazone or bezafibrate was associated with a return to baseline level or higher, in all 3 examined genes. These findings provide evidence for a transcriptional or pre-transcriptional basis of impaired mitochondrial function (attenuated capacity for the dismutation of ROS and diminished insulin sensitivity, reversed by the insulin sensitizer, rosiglitazone) in rats with NAFLD. Thus, the post-treatment amelioration of hepatic steatosis in Ackerman et al’s [28] study may be attributable to the restoration of the hepatic gene levels to their pre-morbid state.

Our study has several limitations. The number of rats in each group was relatively small. The original experiments included a larger sample size (controls −11, FED- 10, FED and rosiglitazone- 9, FED and bezafibrate −10) [28,29], however, the livers of some of the rats were already spent for previously described analyses and only 5 or 6 per group were left for current analyses. Nonetheless, there were no intra-group significant differences in all parameters examined, thus strengthening the validity of our results.

The analyzed samples in the current study were archived from previous studies. None of the frozen liver specimens stored in the laboratory had been subjected to thawing and refreezing. These specimens were still viable for analysis through molecular biology techniques, even though they are known for being highly sensitive to organic degradation. The consistency of the results of the analyses conducted in our laboratory supports our confidence in the reliability of the techniques and the preservation of the frozen tissue.

The results reported in the study relate to one point in time: termination of the experiment (animals sacrifice). No liver samples (biopsies) were taken while the animals were alive. There were no time response or dose response samples relating to the administration of rosiglitazone or bezafibrate.

The FED rat model of NAFLD was imperfect because the rats did not gain weight but nevertheless showed other basic and prominent characteristics of the metabolic syndrome.

## Conclusion

In FED rats with NAFLD, the reduced hepatic expression might be associated with diminished antioxidant capacity and alteration of peroxisomal fatty acid metabolism. All three genes studied are involved in the pathogenesis of NAFLD. Our results imply that the beneficial therapeutic effects of PPAR agonists in patients with NAFLD may be attributed to reversing and normalizing levels of the relevant hepatic genes.

## Competing interests

The authors declare that they have no competing interests or non-financial competing interests.

## Authors’ contributions

HS-W, EH and ZB-Ari made substantial contributions to conception and design, participated in the drafting of the manuscript. MC, YC, SY and AL made substantial contributions to the acquisition of data. EG and ZA gave final approval of the version to be published. All authors read and approved the final manuscript.
